# The University College London/Medical Research Council/National Institute of Health Research-Health Technology Assessment PROMIS Trial: An Update

**DOI:** 10.1016/j.euf.2015.04.007

**Published:** 2015-09

**Authors:** Ahmed El-Shater Bosaily, Christopher Parker, Louise C. Brown, Rhian Gabe, Richard G. Hindley, Richard Kaplan, Mark Emberton, Hashim U. Ahmed, on behalf of the PROMIS Group

**Affiliations:** a Division of Surgery and Interventional Science, University College London, London, UK; b Department of Urology, UCLH NHS Foundation Trust, London, UK; c Department of Academic Urology, Royal Marsden Hospital, Sutton, UK; d MRC Clinical Trials Unit, University College London, London, UK; e Department of Health Sciences, University of York, York, UK; f Department of Urology, Hampshire Hospitals NHS Foundation Trust, Basingstoke, UK

Over the last 10 yr, multiparametric magnetic resonance imaging (mp-MRI) has been suggested as a diagnostic test capable of detecting and ruling out clinically significant gland-confined prostate cancer before biopsy. The poor methodologic quality of many initial studies [1], [2] led to the need for a large robust programme of research to evaluate its clinical validity and utility within the pathway [3]. The PICTURE [4] and Prostate MR Imaging Study (PROMIS) [3] trials met this research need.

Although there has been significant improvement in the literature on mp-MRI, there remain limitations when evaluating clinical validity (sensitivity, specificity, and positive and negative predictive values). For instance, many recent publications have limited sample sizes [5], [6], [7], [8], [9], [10] with no a prior power calculations. Many used histologic reference standards such as transrectal ultrasound (TRUS) biopsy [11], [12], [13], MRI-guided biopsy, and/or limited sampling with the biopsy operators unblinded to the MRI results [10], [14], [15]. Other studies only triggered biopsies when there was an abnormality on mp-MRI [5], [7], [8], [9]. This limits the ability to comment on negative predictive value and introduces operator bias. These factors, among others such as changing technology, reader variability, and variable mp-MRI practice and protocols, contributed to the variability of results on meta-analysis [16], [17].

PROMIS [3] is a multicentre paired-cohort diagnostic trial funded by the National Institute for Health Research Health Technology Assessment and Prostate Cancer UK. A total of 714 biopsy-naïve patients are undergoing optimised guideline-compliant [18] mp-MRI followed by combined 5-mm transperineal mapping biopsies and a 12-core TRUS biopsy under general anaesthetic in the same setting.

PROMIS will provide level 1b evidence [19] on the accuracy of mp-MRI that overcomes the limitations of the current literature [3]. PROMIS will assess the costs and cost-benefit of a prebiopsy MRI pathway (Fig. 1), as well as interobserver variability. Among other outcomes biobanking of serum, plasma, whole blood, and urine will provide samples for the development and validation of biochemical markers.

This diagnostic trial set up in multiple centres proved to be a challenge, with many unforeseen obstacles.

• *Scanner set-up and qualification:* PROMIS uses 1.5-T scanners, the most readily available in most centres. Each scanner had to be optimised in each centre in conjunction with the lead centre in an iterative manner over sometimes weeks and months. We noted significant differences between scanners from the same manufacturer, and even scanners of the same model. These differences required further changes to imaging parameters to remain compliant with international guidelines; one scanner by a certain manufacturer had to be excluded from the study as we were unable to obtain acceptable diffusion sequences after several iterations. These factors will allow us to assess how variations in scanner make, model, and condition may impact on subsequent diffusion and dissemination within the UK and beyond.
• *Lack of essential MRI and interventional ultrasound skills:* To assure correct implementation of a standardised operating procedure in each trial intervention, the lead radiologist formally trained the participating radiologists and held several formal and informal meetings since trial inception to maintain quality control and address issues as they arose. Similarly, each site was required to observe the combined biopsy procedure at trained sites and subsequently required supervision of the conduct of the biopsies.
• *Running a surgical trial:* As a surgical interventional study, issues with respect to resource use arose. First, the mp-MRI standardisation process revealed that some centres were not using gadolinium dynamic contrast enhancement, and the additional 15 min of scan time impacted on scanner capacity. Second, the combined biopsy procedure required between 60 and 90 min of operating theatre time. Third, with a growing body of literature pointing to the positive role of mp-MRI, there were an increasing number of physicians and patients lacking equipoise for the trial.

PROMIS recruitment is on track, with only a 6-mo extension to our original predicted end date. From 11 centres across the UK, 578 patients have been recruited and 98 have withdrawn up to April 2015. Recruitment is due to end in October/November 2015.

PROMIS will provide an once-in-a-lifetime opportunity to evaluate the diagnostic accuracy of mp-MRI based on an adequately large sample size and a highly accurate reference test in comparison to standard TRUS biopsy. The biobank of tissue, imaging data, serum, and urine will offer a uniquely validated data set for further translational research.

***Conflicts of interest:*** Hashim Ahmed receives trial funding from SonoCare Medical, AngioDynamics, and Trod Medical, and a travel allowance from SonoCare Medical. Mark Emberton owns shares in Nuada Medical; is a consultant for Steba Biotech and GlaxoSmithKline; receives trial funding from GlaxoSmithKline, Steba Biotech, SonaCare Medical, AngioDynamics, and Trod Medical; and receives travel funding from Sanofi Aventis, Astellas, GlaxoSmithKline, and SonaCare Medical. Alex Kirkham and Alex Freeman own shares in Nuada Medical. The remaining authors have nothing to disclose.  

***Acknowledgments:*** We acknowledge trial funding from the National Institute of Health Research – Health Technology Assessment (Project 09/22/67) and from Prostate Cancer UK for collection and processing blood and urine for the translational aspect of PROMIS (PROMIS-T). The project is also supported and partially funded by UCLH/UCL Biomedical Research Centre and The Royal Marsden and Institute for Cancer Research Biomedical Research Centre and is coordinated by the Medical Research Council Clinical Trials Unit (MRC CTU) at UCL. It is sponsored by University College London (UCL).  

***Department of Health Disclaimer:*** The views and opinions expressed herein are those of the authors and do not necessarily reflect those of the Health Technology Assessment programme, the National Institute of Health Research, the National Health Service, or the Department of Health.

## Figures and Tables

**Fig. 1:**
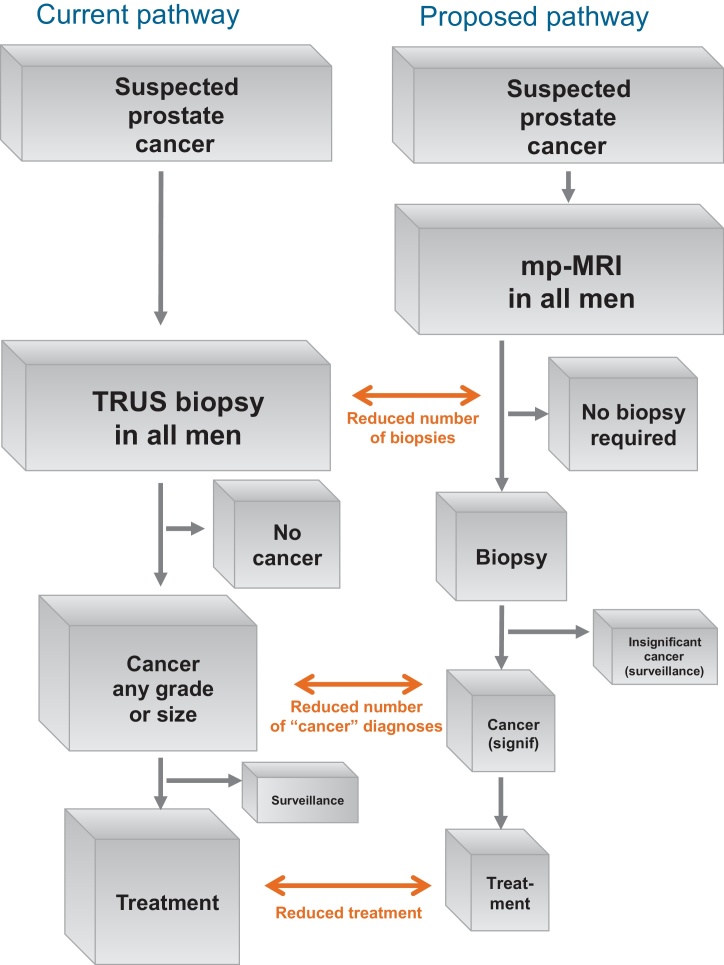
Potential clinical impact of the PROMIS study. mp-MRI = multiparametric magnetic resonance imaging; TRUS = transrectal ultrasound; signif = significant.

## References

[1] Kirkham A.P., Emberton M., Allen C. (2006). How good is MRI at detecting and characterising cancer within the prostate?. Eur Urol.

[2] Ahmed H.U., Kirkham A., Arya M. (2009). Is it time to consider a role for MRI before prostate biopsy?. Nat Rev Clin Oncol.

[3] El-Shater Bosaily A., Parker C., Brown L.C. (2015). PROMIS—prostate MR imaging study: a paired validating cohort study evaluating the role of multi-parametric MRI in men with clinical suspicion of prostate cancer. Contemp Clin Trials.

[4] Simmons L.A., Ahmed H.U., Moore C.M. (2014). The PICTURE study—prostate imaging (multi-parametric MRI and prostate HistoScanning) compared to transperineal ultrasound guided biopsy for significant prostate cancer risk evaluation. Contemp Clin Trials.

[5] Abd-Alazeez M., Kirkham A., Ahmed H.U. (2014). Performance of multiparametric MRI in men at risk of prostate cancer before the first biopsy: a paired validating cohort study using template prostate mapping biopsies as the reference standard. Prostate Cancer Prostatic Dis.

[6] Chamie K., Sonn G.A., Finley D.S. (2014). The role of magnetic resonance imaging in delineating clinically significant prostate cancer. Urology.

[7] Sonn G.A., Chang E., Natarajan S. (2014). Value of targeted prostate biopsy using magnetic resonance-ultrasound fusion in men with prior negative biopsy and elevated prostate-specific antigen. Eur Urol.

[8] Abd-Alazeez M., Ahmed H.U., Arya M. (2014). The accuracy of multiparametric MRI in men with negative biopsy and elevated PSA level—can it rule out clinically significant prostate cancer?. Urol Oncol.

[9] Arumainayagam N., Ahmed H.U., Moore C.M. (2013). Multiparametric MR imaging for detection of clinically significant prostate cancer: a validation cohort study with transperineal template prostate mapping as the reference standard. Radiology.

[10] Thompson J.E., Moses D., Shnier R. (2014). Multiparametric magnetic resonance imaging guided diagnostic biopsy detects significant prostate cancer and could reduce unnecessary biopsies and over detection: a prospective study. J Urol.

[11] Tamada T., Sone T., Higashi H. (2011). Prostate cancer detection in patients with total serum prostate-specific antigen levels of 4–10 ng/ml: diagnostic efficacy of diffusion-weighted imaging, dynamic contrast-enhanced MRI, and T2-weighted imaging. Am J Roentgenol.

[12] Vilanova J.C., Barcelo-Vidal C., Comet J. (2011). Usefulness of prebiopsy multifunctional and morphologic MRI combined with free-to-total prostate-specific antigen ratio in the detection of prostate cancer. Am J Roentgenol.

[13] Iwazawa J., Mitani T., Sassa S., Ohue S. (2011). Prostate cancer detection with MRI: is dynamic contrast-enhanced imaging necessary in addition to diffusion-weighted imaging?. Diagn Interv Radiol.

[14] Rais-Bahrami S., Siddiqui M.M., Turkbey B. (2013). Utility of multiparametric magnetic resonance imaging suspicion levels for detecting prostate cancer. J Urol..

[15] Rouse P., Shaw G., Ahmed H.U., Freeman A., Allen C., Emberton M. (2011). Multi-parametric magnetic resonance imaging to rule-in and rule-out clinically important prostate cancer in men at risk: a cohort study. Urol Int.

[16] Futterer JJ, Briganti A, De Visschere P, et al. Can clinically significant prostate cancer be detected with multiparametric magnetic resonance imaging? A systematic review of the literature. Eur Urol. In press. http://dx.doi.org/10.1016/j.eururo.2015.01.013 10.1016/j.eururo.2015.01.013 25656808

[17] Rooij Md, Hamoen E.H.J., Fütterer J.J., Barentsz J.O., Rovers M.M. (2014). Accuracy of multiparametric MRI for prostate cancer detection: a meta-analysis. Am J Roentgenol.

[18] Barentsz J., Richenberg J., Clements R. (2012). ESUR prostate MR guidelines 2012. Eur Radiol.

[19] Centre for Evidence-Based Medicine (2013). Levels of evidence 1.

