# Supplementary material for: The University College London/Medical Research Council/National Institute of Health Research-Health Technology Assessment PROMIS Trial: An Update
Source: Eur Urol Focus. 2015 Sep;1(2):212–4. doi: 10.1016/j.euf.2015.04.007 (PMC4694096; doi:10.1016/j.euf.2015.04.007)
Supplement: Supplementary file 1 [file mmc1.doc]

**Appendix 1 - The PROMIS Study Group**

**Trial Sponsor:** University College London (UCL)

**Trial Coordination:** Medical Research Council Clinical Trial Unit (MRC CTU) at UCL

**Funders:** National Institute for Health Research (NIHR) HealthTechnology Assessment (HTA) & Prostate Cancer UK

**Trial Management Group:**

Professor Mark Emberton (Chief Investigator) UCLH (Urologist)

Mr Hashim Ahmed (Co-Investigator) UCLH (Urologist)

Dr Ahmed El-Shater Bosaily UCLH (Clinical Fellow)

Dr Alex Kirkham UCLH (Radiologist)

Dr Alex Freeman UCLH (Pathologist)

Dr Charles Jameson UCLH (Pathologist)

Mr Richard Hindley (Co-Investigator) Basingstoke (Urologist)

Dr Christopher Parker (Co-Investigator) Royal Marsden

(Translational Research)

Professor Colin Cooper Royal Marsden

(Translational Research)

Robert Oldroyd Patient representative

Professor Richard Kaplan MRC CTU

(Programme Lead/Oncologist)

Dr Louise Brown MRC CTU

(Project Lead/statistician)

Dr Rhian Gabe University of York

(Statistician)

Dr Yolanda Collaco-Moraes MRC CTU

(Clinical Operations Manager)

Cybil Adusei, Katie Ward MRC CTU (Trial Managers)

Sophie Stewart, Katie Thompson

Claire Mulrenan, Hannah Gardner, Sara Smart MRC CTU (Data Managers)

Carlos Diaz-Montana MRC CTU (Data programmer)

Dr Chris Coyle MRC CTU (Clinical Fellow)

Professor Mark Sculpher University of York

(Health Economics)

Dr Rita Faria University of York

(Health Economics)

**Trial Steering Committee (also act as Data Monitoring Committee):**

Dr David Guthrie (Chair) Derbyshire Royal Infirmary

(Oncologist)

Professor John Chester University of Cardiff

(Oncologist)

Professor Richard Cowan Christie Hospital Manchester

(Oncologist)

Professor Michael Jewitt University of Toronto

(Urologist)

**Participating Centres:**

*University College London Hospital*: H. Ahmed (PI), A. El-Shater Bosaily, M. Emberton, A. Kirkham, S. Punwani, A. Freeman, C. Jameson, M. Hung, J. Coe, R. Scott.

*Basingstoke and North Hampshire Hospital*: R. Hindley (PI), D. Peppercorn, A. Thrower, H. El-Mahallawi, A. Mustajab, H. Alkhazaraji, A. Edwards, J. Smith.

*Charing Cross Hospital, Imperial College London*: M. Winkler (PI), T. Barwick, V. Stewart, L. Honeyfield, N. Qazi, B. Statton, N. Ngo, K. Ansu, S. Edwards, E. Temple.

*Musgrove Park Hospital*: N. Burns-Cox (PI), K. Gordon, A. Birring, A. Maccormick, P. Burn, D. Paterson, H. Routley.

*Maidstone Hospital*: A. Henderson (PI), S. Ghosh, P. McMillan, J. James, G. Russell, E. Bernsten, R. Casey, T. Nolan, D. Day.

*Southmead Hospital, Bristol*: R. Persad (PI), M. Elmahdy, S. Pandian, D. Gillatt, J. Ash-Miles, M. Sohail, C. Shiridzinomwa, A. Treasure.

*The Whittington Hospital*: M. Ghei (PI), T. Shah, J. Kumaradevan, A. Trinidade, R. Katz, D. Arul, L. Harbin, V. Conteh, A. Verjee.

*Royal Hallamshire Hospital, Sheffield*: D. Rosario (PI), J. Catto, F. Salim, S. Morgan, J. Howson.

*Wrexham Maelor Hospital*: I. Shergill (PI), S. Agarwal, K. Pradeep, S. Ackerley.

*Frimley Park Hospital:* S. Bott (PI), H. Evans, G. Kousparos, A.M. Silvanto, A. Mann, J. Amero, A. Pilcher.

*Southampton General Hospital*: T. Dudderidge (PI), J. Smart, K. Tung, H. Markham, A. Lodge.
